# The Successful Treatment of Multi-Resistant Colonized Burns with Large-Area Atmospheric Cold Plasma Therapy and Dermis Substitute Matrix—A Case Report

**DOI:** 10.3390/ebj5030025

**Published:** 2024-08-26

**Authors:** Moritz R. Milewski, Frederik Schlottmann, Vincent März, Thorben Dieck, Peter M. Vogt

**Affiliations:** Department of Plastic, Aesthetic, Hand and Reconstructive Surgery, Hannover Medical School, Carl-Neuberg-Strasse 1, 30625 Hannover, Germany; schlottmann.frederik@mh-hannover.de (F.S.); maerz.vincent@mh-hannover.de (V.M.); dieck.thorben@mh-hannover.de (T.D.); vogt.peter@mh-hannover.de (P.M.V.)

**Keywords:** burns, burn units, cold plasma therapy, dermal skin substitute, intensive care units, meek micrograft, plastic surgery procedures, skin substitutes, skin transplantation

## Abstract

The treatment of severe burn injuries, which occur particularly in the context of armed conflicts, is based on a multimodal treatment concept. In addition to complex intensive care therapy, the surgical reconstruction options of plastic surgery and typical antiseptic wound treatment are the main focuses. In recent years, atmospheric cold plasma therapy (ACPT) has also become established for topical, antiseptic wound treatment and for the optimization of re-epithelialization. This case report shows a successful treatment of extensive burn injuries using dermal skin substitute matrix and topical treatment with a large-area cold plasma device to control multi-resistant pathogen colonization. This case report illustrates the importance of ACPT in burn surgery. However, larger case series and randomized controlled trials in specialized centers are needed to assess its place in future clinical practice.

## 1. Introduction

In recent years, the centuries-old technology of atmospheric cold plasma (ACPT) has once again come into focus for wound therapy [1]. The technical background of ACPT involves understanding the principles of plasma generation, the properties of ACPT and its interactions with biological tissues. The basics of non-thermal cold plasma were first described by William Crookes in 1879 by experimentally ionizing gas in an electrical discharge tube by the application of high voltage, but the current term of plasma was initiated as late as 1927 by Irvin Langmuir [1,2]. Plasma is often referred to as the fourth state of matter, consisting of a partially ionized gas with equal numbers of positive ions and free electrons [3]. To generate plasma, energy is applied to a gas, causing ionization [4]. There are several methods to generate plasma, including dielectric barrier discharge, corona discharge and radio frequency discharge [5,6]. ACPT is a safe and efficient therapeutic tool in clinical practice, as shown by various preclinical and clinical studies [7]. Current applications in medicine range from the treatment of chronic wounds or skin infections to targeted cancer therapy in oncology and various applications in dentistry [7,8]. The therapeutic effects of ACPT are primarily due to the interaction of reactive oxygen and nitrogen species (RONS) with cells and tissues [9,10]. These interactions include antimicrobial action, topical cancer therapy and wound healing. However, regarding burn wound care, the ACPT data are limited to isolated case reports or series, with no study covering the topic of war burns.

In the context of current armed conflicts, burn surgery is experiencing a renaissance with a significant increase in patients [11]. As part of the broader humanitarian support, German hospitals have been treating Ukrainian soldiers wounded in the conflict with Russia, offering advanced burn care and rehabilitation [12]. War invalids from Ukraine are transferred to Germany according to a fixed distribution system, known as the cloverleaf system [13]. The treatment of severe burn injuries occurs in specialized burn centers equipped with highly advanced technology and personnel [14]. Soldiers often arrive with delays after passing through various care points, starting with emergency care in frontline field hospitals, followed by treatment in other facilities, and finally being airlifted to Germany.

Thus, we treat not fresh burns, which are typically sterile, but wounds already contaminated with bacteria. These infections often involve problematic or hospital-acquired pathogens like *Acinetobacter* and *Pseudomonas* spp., which exhibit high levels of antibiotic resistance [15]. This poses a significant challenge, as we frequently encounter cases where even reserve antibiotics fail. Our approach to managing these complex wound situations necessitates a multimodal therapy concept beyond the classical wound debridement and split-thickness skin grafting [16]. While these remain the foundation, we incorporate advanced treatments to address the specific needs of our patients. NovoSorb^®^ Biodegradable Temporizing Matrix (BTM) (PolyNovo, Carlsbad, CA, USA) is one example, used for its effectiveness in reconstructing deep dermal burns, even in the presence of resistant bacteria [17,18]. BTM, a fully synthetic dermal skin substitute, consists of a two-layered biodegradable polyurethane foam with a temporary non-degradable sealing membrane, applied in a two-staged procedure often combined with vacuum therapy [19,20]. Additionally, phage therapy has been utilized in cases with multi-resistant pathogen colonization [18]. This approach, which involves using bacteriophages to target and destroy specific bacteria, goes far beyond classical standard therapy. The use of phages has shown promise in managing infections that are unresponsive to conventional antibiotics, providing an innovative solution to a critical problem [21].

Building on this multimodal approach, we are now implementing ACPT to further enhance our treatment arsenal [1]. ACPT generates RONS, which have potent antimicrobial effects, crucial for our patient cohort [9,10]. ACPT is a safe and efficient tool in clinical practice, demonstrating effectiveness in preclinical and clinical studies [7]. It has been used in diabetic wounds, which are often difficult to treat due to poor vascularization [22]. The non-thermal nature of ACPT ensures that it can be applied to sensitive tissue without causing local damage or significant discomfort [1]. ACPT’s antimicrobial properties are due to the interaction of RONS with cell membranes, proteins, or nucleic acids of pathogens, leading to their inactivation [10,23]. Studies have shown that ACPT can significantly reduce the microbial burden on wounds, preventing infections that complicate healing [7,8]. This is particularly beneficial for treating burn wounds colonized by multidrug-resistant pathogens [24]. Additionally, ACPT promotes wound healing by stimulating cell proliferation, migration, and differentiation. The RONS modulate signaling pathways involved in tissue regeneration and reduce inflammation, enhancing microcirculation and cellular responses [25]. Various studies have demonstrated that ACPT treatment can improve wound closure rates, reduce inflammation, and enhance tissue regeneration [23,25]. For instance, ACPT has shown an increased expression of vascular endothelial growth factor (VEGF) and other growth factors that are key molecules for neo-angiogenesis and tissue regeneration [25].

The introduction of the CPT^®^cube and CPT^®^patch by COLDPLASMATECH (Greifswald, Germany) provides a new opportunity to treating extensive wounds with an adaptable amount of cold plasma that is automatically generated to ensure optimal treatment conditions within minutes of treatment—independent of the size and depth of the area treated [26]. Additionally, this system guarantees treatment consistency and reproducibility, eliminating the effects of user variability. This application method enables the efficient treatment of large surface areas, which is crucial for our patients with extensive burns and high microbial loads. By combining ACPT with our existing therapies, we aim to ensure successful skin graft integration and prevent both local and systemic infections.

Among other successful treatments, this case report highlights the importance of ACPT using the example of a Ukrainian soldier with extensive burn wounds and defect coverage with BTM and meek micrograft transplantation against the backdrop of multi-resistant pathogen colonization. The integration of ACPT into our treatment regimen represents a significant advance in managing these challenging cases.

## 2. Detailed Case Description

The data of the case report at hand are based on operation reports, photo documentation and further available information from the electronic patient file of the hospital information system during the course of treatment in the burn intensive care unit (ICU) of the Department of Plastic, Aesthetic, Hand and Reconstructive Surgery at Hannover Medical School. The patient gave his informed written consent to publish this paper.

A 40-year-old male soldier from the Ukrainian armed forces sustained severe injuries while engaged in combat during the Ukraine–Russia conflict. In February 2024, he was struck by rocket fire. A direct hit near his position resulted in the traumatic amputation of his left lower leg and the ignition of his pants, leading to significant burns. Following stabilization at a front-line primary care hospital, the patient underwent epifascial debridement and amputation of the left lower leg in Kyiv. He was then transferred to our clinic via the national cloverleaf system as described above.

**Figure 1:**
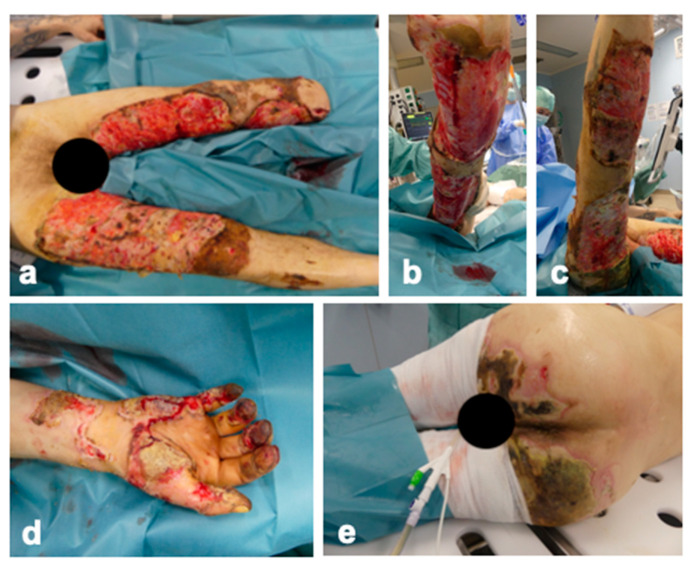
Wound condition on admission to burn intensive care unit. (**a**) General view of the debrided 2b-3 degree burns in the area of the lower extremities from the ventral side; (**b**) dorsal view of the left lower extremity; (**c**) dorsal view of the right lower extremity; (**d**) 2b-3 degree burns in the area of the left hand with necrosis of the little finger; (**e**) untreated third-degree burn on the buttocks.

The patient was admitted to our specialized burn ICU 15 days after the initial injury. This delay in the transfer to our specialized burn unit was critical, as we had anticipated the presence of highly resistant pathogens. On admission to the burn ICU, the patient was awake and cardiopulmonary stable. The patient stated that he was 180 cm tall and weighed 80 kg. Figure 1 shows the wound condition on admission. Under analgesia sedation, adhered wound dressings were removed, revealing extensive epifascial abrasions on both legs (Figure 1a–c) and a mid-tibia level amputation with a high fibula fracture on the left leg (Figure 1a). Second-degree burns affected the right hand, and the distal phalanx of the little right finger was necrotic (Figure 1d). Untreated third-degree burns were noted on the buttocks (Figure 1e). Overall, approximately 23% total body surface area (TBSA) was affected, with an abbreviated burn severity index (ABSI) of 6 points. No previous illnesses were known. Preliminary medical documentation was incomplete and only available in Ukrainian.

Initial microbiological results from the Ukrainian hospital indicated wound colonization with *Bacillus cereus* and *Enterococcus faecalis*. The patient received an intraoperative single-shot calculated antibiotic treatment with piperacillin/tazobactam and vancomycin without postoperative continuation as suggested by our antibiotic stewardship team as an empirical anti-infective therapy based on the previous Ukrainian results. Given the microbiologically and macroscopically contaminated wounds, hydrotherapeutic wound debridement and wound care with aseptic dressings was initially performed. The patient was admitted to the burn ICU spontaneously breathing and with stable circulation. Microbiological samples were taken as part of the initial treatment (day 0). Preventive isolation of the patient was carried out in the burn ICU. To achieve clean wound conditions, operative wound debridement was performed the following day (day 1) using sharp surgical techniques in combination with the ultrasonic-assisted hydro-surgical debridement system Sonoca 300 (Söring GmbH, Quickborn, Germany). Intraoperatively, microbiological samples were again collected to determine the microbial burden. For temporary wound coverage, the application of EpiGARD^®^ (Biovision GmbH, Ilmenau, Germany) as a temporary skin substitute on both legs and polyhexanide gel and fatty gauze on the right hand were performed. After intraoperative temporary wound covering with EpiGARD^®^, daily dressing changes were performed with topical wound disinfection using iodine (Braunol^®^, B. Braun SE, Melsungen, Germany) and octenidine dihydrochloride (octenisept^®^, Schülke & Mayr, Norderstedt, Germany) solutions.

In congruence with our experience in the treatment of other Ukrainian patients, the microbiological findings from day 0 and day 1 showed a mixed flora of multi-resistant pathogens. Table 1 gives an overview of the microbial burden detected on admission and after the first surgery on day 1, as well as the development over the further course of treatment. The microbiological results indicated the colonization with *Acinetobacter baumannii* of all wounds, the nose, throat, and rectal areas, which was of particular concern (Table 1). Blood cultures and urine were found to be sterile on admission. Our experience with Ukrainian soldiers has shown that they were always colonized or infected with these problematic pathogens. Due to operative care, there is a risk of pathogen translocation leading to sepsis, causing previously stable patients to decompensate. Therefore, our goal was to reduce the local bacterial load as much as possible. Only reserve antibiotics like cefiderocol and colistin presented as effective against these germs but these are known to have considerable drawbacks like a significant cost and undesirable drug effects. Thus, we decided to restrict them to inevitable administration, e.g., perioperative treatment and sepsis. Despite considering phage therapy to combat *Acinetobacter baumannii*, we could not proceed due to the unavailability of suitable phages from the National Center for Phage Therapy at Hannover Medical School. Phage therapy has shown promise in managing infections that are unresponsive to conventional antibiotics, providing an innovative solution to a critical problem. However, in this case, the lack of appropriate phages necessitated alternative approaches.

**Figure 2:**
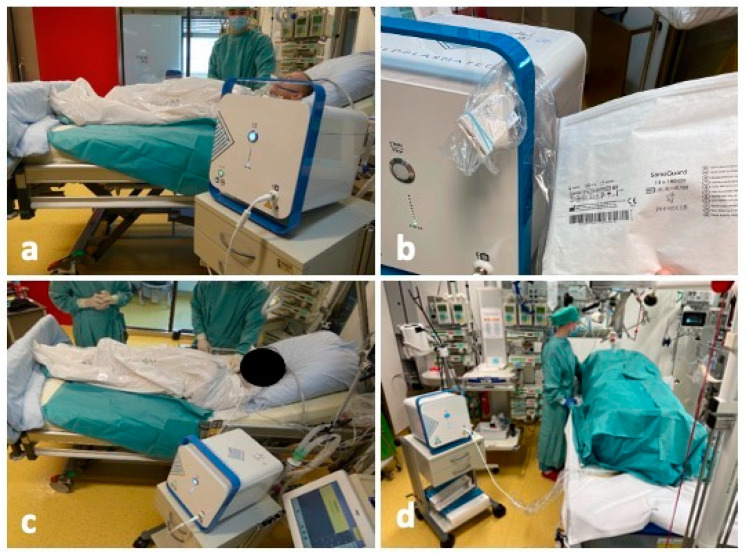
Full body therapy using atmospheric cold plasma. (**a**) Atmospheric cold plasma device of COLDPLASMATECH (Greifswald, Germany); (**b**) the patient was placed in a treatment bag, with the CPT^®^patch left inside to perform three consecutive cycles (altering the 2 min according to the manufacturer’s instructions to 6 min of treatment because of the large area treated) of plasma application using the COLDPLASMATECH device. (**c**) The CPT^®^patch; (**d**) the whole patient is treated with ACPT to reduce microbial load, excluding the nasopharyngeal area.

Consequently, we rapidly initiated ACPT to reduce the microbial load and mitigate the risk of systemic infection. As ACPT has shown promising results in treating chronic wounds with microbial colonization but the treatment area is limited in most devices, we incorporated the cold plasma device from COLDPLASMATECH (COLDPLASMATECH GmbH, Greifswald, Germany) into our treatment regimen (Figure 2a). The treatment was continued daily, with the CPT^®^patch (Figure 2b) used inside an airtight bag to enclose the patient’s entire body, leaving only his head uncovered (Figure 2c). This allowed for extensive application of RONS to all wound areas, spaces, cavities and dressings, which is crucial for comprehensive microbial reduction. To reduce the bacterial load in the nasopharyngeal region, the patient was also completely covered intermittently with drapes and ACPT was applied to the entire patient (Figure 2d).

On day 6, the surgical epifascial debridement of all remaining necrotic areas was conducted, followed by hydro-surgical VERSAJET (Smith & Nephew, London, UK) debridement and temporary coverage with BTM. The BTM was slit or fenestrated in several places to allow fluid drainage and the safe passage of cold plasma. Antibiotics were discontinued after prolonged admission postoperatively, as recommended by our antibiotic stewardship team, while ACPT continued. The patient remained stable and BTM integration proceeded despite the formation of pus draining through the breaches containing massive amounts of *Acinetobacter baumannii*. This allowed for the split-thickness skin grafting of the right leg using the meek micrograft technique on day 29. The patient was positioned on an air-fluidized bed postoperatively to reduce shear forces and pressure on the grafted areas.

**Figure 3:**
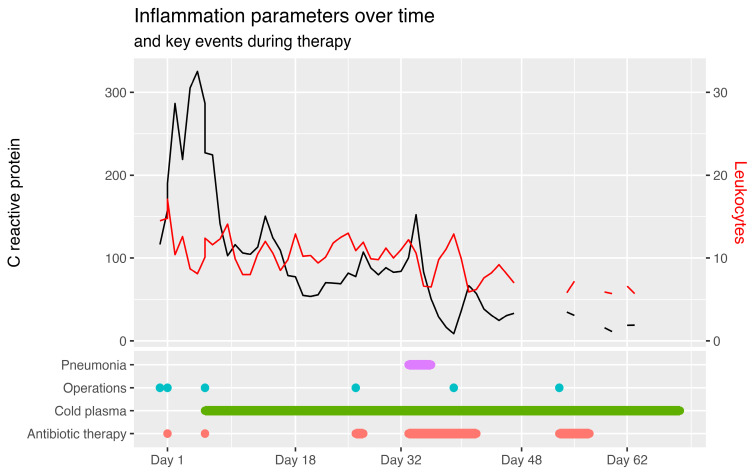
Summary of infection parameters and treatment as well as clinical milestones over time. C reactive protein in mg/L and leukocyte level in 1000/µL. Events such as operations, pneumonia, cold plasma therapy and antibiotic treatment primarily account for the inflammation parameters shown above.

On day 33, the patient developed sudden delirium, a rise in serum inflammation parameters, opaque and yellowish sputum as well as infiltrates in the thoracic X ray which were interpreted as sepsis from pneumonia caused by *Acinetobacter baumannii*, requiring the renewed administration of cefiderocol and meropenem. Figure 3 shows the course of the infection parameters over the entire course of treatment.

**Figure 4:**
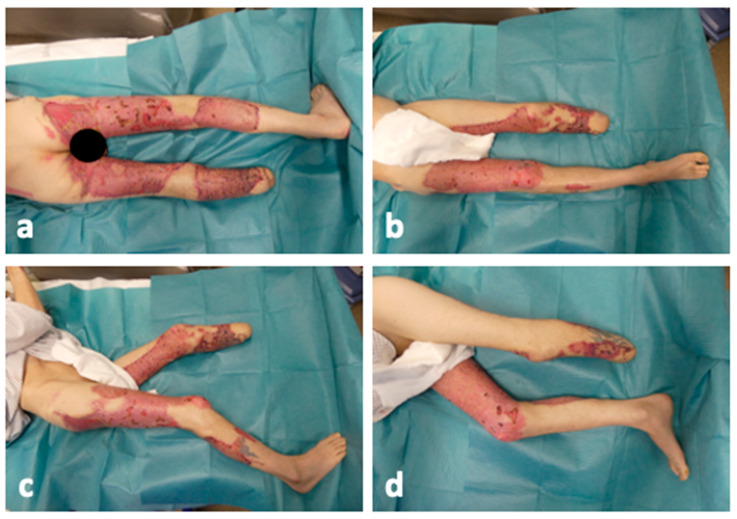
The lower extremity from every angle on day 70 after BTM application, ACPT and meek micrografting. (**a**) Dorsal view of the wounds after 70 days; (**b**) ventral view of the wounds after 70 days; (**c**) right-sided view of the wounds after 70 days; (**d**) left-sided view of the wounds after 70 days. The treatment concept with ACPT for microbial reduction and BTM and meek micrografting for defect coverage led to almost complete wound consolidation after 70 days.

Following the total regression of sepsis at day 36, further split-thickness skin grafting and debridement procedures were successfully performed on day 38, extending the administration of cefiderocol and meropenem for two days postoperatively. On day 53, a re-transplantation of the gluteal region using meek micrografting was necessary. Hereafter, an approximately 90% coverage of the burned areas was achieved. The wound healing process was not yet fully completed at the time of this case report, but it had progressed significantly, as shown in Figure 4. The smaller residual defects were subjected to secondary wound healing.

This multimodal and complex therapeutic approach, integrating advanced techniques such as BTM and ACPT, aims to ensure successful wound healing and graft integration while preventing systemic infections. Daily treatment with the cold plasma device helped to manage the microbial burden effectively, reducing the risk of pathogen translocation and subsequent sepsis.

## 3. Discussion

The presented case illustrates the complex management of severe burn injuries complicated by multi-drug resistant infections. The integration of novel therapies such as ACPT, alongside traditional wound care and selective antibiotic use, highlights the potential for innovative approaches in managing challenging cases in the post-antibiotic era.

The transfer of Ukrainian patients to Germany can now be considered a standardized procedure [13]. However, there are challenges that need to be overcome in their future treatment. Once the acute treatment has been completed, multidisciplinary rehabilitation treatment takes place, beginning in the acute hospital and continuing in outpatient and inpatient rehabilitation areas [27]. A significant problem remains in the uncovered costs for rehabilitation and nursing home accommodation for patients who are seriously injured, cannot be rehabilitated and cannot return to Ukraine. Furthermore, there is a lack of comprehensive outpatient care structures and interpreters for Ukrainian patients, whose costs have to be borne and whose payer is often unclear [28].

To our knowledge, this paper mentioning ACPT is among the first for burn wound treatment, especially in combination with BTM. Numerous studies with BTM have been conducted to investigate the safety and ability for permanent wound closure in combination with autologous split-thickness skin grafts in a two-stage surgical procedure in sheep, pigs and humans [20]. However, a disadvantage was found to be the period of up to 3 weeks for integration and vascularization of BTM, resulting in prolonged hospitalization times [17]. As shown by the present case report, BTM has once again proven its versatile properties and application in microbial-colonized wounds as described elsewhere [17,18]. Regarding the combination of BTM with meek micrografting, the results of the presented case report are in line with the only available case series in the literature, showing reliable aesthetic and functional results [19].

Despite medical efforts, wound infections still pose a major challenge when treating burn patients in a burn ICU, especially with pathogens like *Pseudomonas aeruginosa*, *Acinetobacter baumannii* and others [29,30]. The major problem leading to increased mortality lies in the emergence of drug-resistant strains [29,30]. Ziuzina et al. explored the ability of cold plasma to inactivate bacterial biofilms and reduce quorum sensing-regulated virulence factors [31]. ACPT has the ability to reduce microbial load, making it a good option to replace antibiotics and combat bacterial strains with increasing antibiotic resistance [32]. Successful case reports using ACPT demonstrated reduced the microbial burden and improved the healing capacities of wounds colonized with *Pseudomonas aeruginosa* and multidrug-resistant *Staphylococcus aureus* (MRSA) [33,34]. Through the application of 180 s of ACPT to MRSA, the authors observed significantly decreased biomass and susceptible biofilms after 72 h [34]. Other studies described the successful decolonization of MRSA, *Staphylococcus aureus* and *Escherichia coli* using ACPT in a porcine skin model in vitro [35], as well as a reduction in the need for antibiotics in the treatment arm compared to standard wound therapy, from 23% to 4% [26]. Bayliss et al. even found the restoration of antibiotic sensitivity in MRSA following treatment with non-thermal atmospheric gas plasma [36].

The first randomized controlled trials showed a reduced wound surface and a shortened time to wound closure, independent of background disinfection in the treatment of chronic diabetic ulcers [22]. Souza et al. investigated the effect of argon atmospheric plasma on burn wound healing. They found that cold plasma promotes wound healing by stimulating the inflammatory response and controlling the redox state. The study shows that plasma influences the release of cytokines and growth factors, leading to accelerated healing [37], whereas Duchesne et al. focused on the modulation of endothelial nitric oxide synthase (eNOS) by cold atmospheric plasma and its impact on burn wound neovascularization. They demonstrated that cold plasma enhances eNOS signaling and promotes the formation of new blood vessels, supporting the healing of burn wounds [38].

Other studies showed the potential of ACPT mediated by RONS as therapeutic mediators in inactivating viruses such as SARS-CoV-2 [39]. Furthermore, an animal study in burned mice showed that wounds treated with ACPT healed faster than the controls based on the enhanced expression of the dermal–epidermal junctions’ components and increased collagen type I expression [40].

However, little is known about the effect of ACPT on *Acinetobacter baumannii*. One study reported that increased *Acinetobacter baumannii* biofilm reduced the efficacy of antimicrobial properties of ACPT, resulting in greater tolerance to plasma exposure [41]. Therefore, the early and consistent application of ACPT appears to be promising in cases of wound colonization with *Acinetobacter baumannii*, as demonstrated in this case report. ACPT has shown promising results in the treatment of burn wounds by leveraging its antimicrobial properties and ability to promote wound healing [24], but the published literature is still limited to preclinical studies or isolated case series. For example, ACPT has been described as a promising alternative in the case of split skin graft failure in a 42-year-old female patient with extensive wound healing disorders [42]. Taking the findings of all published studies together, ACPT is a versatile option to reduce microbial burden in wound therapy and improve wound healing.

In our multimodal therapy approach, the effect of cold plasma cannot be viewed in isolation. We did not conduct a quantitative analysis of microbial load, and therefore, the plasma effect cannot be directly inferred from our data. This case report merely describes an instance where, compared to similar cases where we faced extreme difficulties with multi-resistant pathogens, the use of ACPT appeared to have mitigated these issues. This can only serve as a suggestion for further investigation, and no causality can be derived from this single case.

Some studies reported that the effect of plasma application can vary widely even within a one-centimeter difference in human tissue, and hypothetically minimal negative effects at the molecular level could not be excluded when using handheld devices like plasma jets [43]. All these findings and possibilities are subject to ongoing studies, and current results suggest that the unproven adverse effects are outweighed by ACPT’s many benefits. Furthermore, these adverse results could not be reproduced in this case report with the automatic device used. Rather, an extensive application to the entire body of the patient appears to develop a comprehensive effect of the ACPT, as can be seen from the increasing wound consolidation and decreasing bacterial load. Compared to other plasma devices certified for clinical practice, such as the kINPen^®^ MED plasma-pen (INP Greifswald/neoplas tools GmbH, Greifswald, Germany), the PlasmaDerm^®^ device (CINOGY Technologies GmbH, Duderstadt, Germany) and the SteriPlas plasma torch device (Adtec Ltd., London, UK), the plasma device used in the presented study (COLDPLASMATECH, Greifswald, Germany) showed comparable and reliable results regarding antimicrobial effects and improved wound healing [44]. Research into ACPT is an ongoing process with studies focusing on optimizing plasma sources, understanding the mechanism of action and expanding clinical applications [45].

In modern burn treatment approaches, pathogen-specific phages are now used for targeted topical treatment as part of isolated therapeutic trials [18]. However, as can be seen in this case, phages are not available or clinically applicable in all cases. Modern treatment approaches are therefore needed to apply typical and systematic anti-infective therapeutic approaches, particularly in the case of complex multi-resistant bacterial colonization. Regarding the microbial burden of the burn wounds in the current case report, the results are in line with the published literature. Of great concern are carbapenemase-producing Gram-negative bacteria (CPGN), such as *Acinetobacter baumannii* and *Klebsiella pneumoniae*, as seen in epidemiological studies among refugees and war-wounded Ukrainians [15,29]. The rising prevalence has a direct influence on the patients’ treatment and the German health system, such as infection prevention measures, a higher number of isolations, additional microbiological testing and overall organization within the hospital [15].

The present case report can be seen as a valuable complement to the published literature. Overall, ACPT leverages the unique properties of non-thermal plasma that lead to beneficial therapeutic effects such as antimicrobial action, wound healing and cancer treatment, making it a versatile and future-oriented therapeutic tool in modern medicine. As there is a lack of randomized controlled trials as well as sufficient meta-analysis, further studies are needed in the near future to evaluate its potential in clinical application. Expertise should be bundled in comprehensive centers that use ACPT as part of the clinical routine.

## 4. Conclusions

Automated large-scale ACPT represents a novel and effective approach to managing burn wounds, combining potent antimicrobial action with enhanced healing capabilities. This dual-action makes it a valuable addition to the current therapeutic arsenal for burn care. The present case report was able to illustrate the importance in the treatment of multi-resistant colonized complex burn wounds and prove a safe, patient individual, time-effective application. Further research and clinical trials will help optimize its application and establish standardized treatment protocols. Bundling expertise in specialized centers seems promising for future applications.

## Figures and Tables

**Table 1:** Microbiological results of wound swabs shown at respective days after admission. Ordinal data about the bacterial load are provided by our department of microbiology and abbreviated as follows: low bacterial load (+), medium bacterial load (++), high bacterial load (+++). The detection of a specific bacterial species without any information about the bacterial load is referred to as “positive”. The blank cells of the table represent no detection of the respective bacterial species.

Location	Day	*Acinetobacter baumannii* 4-MRGN	*Escherichia coli*	*Enterobacter cloacae* 3-MRGN	Vancomycin Resistant *Enterococcus* Faecium	*Pseudomonas aeruginosa* 4-MRGN	*Corynebacterium striatum*	Staphylococcus Coagulase Negative
Buttocks	0	+++	+					
Buttocks	11	++			++		++	
Buttocks	67						++	++
Left leg	0	++						
Left leg	0	+++						
Left leg	1	+++		positive				
Left leg	4	+++		++				
Left leg	11	++					++	
Left leg	67							+
Rectum	0	+++	positive		positive			
Rectum	4	positive						
Rectum	11	positive		positive	positive	positive		
Right arm	4	++						
Right arm	11				+			+
Right arm	67						++	
Right leg	0	++						
Right leg	1	+++						
Right leg	4	+++		++				
Right leg	11	++			+		++	
Right leg	67	+				+	++	++

## Data Availability

The original contributions presented in the study are included in the article; further inquiries can be directed to the corresponding author.
